# Offering HIV testing in an emergency admission unit in Newcastle upon Tyne, UK — a pilot audit study

**DOI:** 10.1186/1758-2652-13-S4-P168

**Published:** 2010-11-08

**Authors:** S Ellis, L Graham, DA Price, ELC Ong

**Affiliations:** 1 Royal Victoria Infirmary, Department of Infection and Tropical Medicine, Newcastle upon Tyne, UK

## Background

The UK National Guidelines for HIV Testing 2008 recommends that HIV testing should be offered to all general medical admissions where the reported prevalence of HIV is >2/1000 (1). We have previously reported that in Newcastle, 72% of new diagnoses in 2007 were late presenters (2) compared to 55% nationally.

## Purpose of the study

A prospective audit was undertaken offering HIV testing to all general medical admissions attending the Emergency Assessment Unit (EAU) in Newcastle to assess feasibility, acceptability and point prevalence.

## Methods

All patients attending EAU with capacity to verbal consent were offered HIV testing during two block periods of 6 and 11 weeks in 2009/10. The first period was physician led, the second physician-assistant led. Training was undertaken and led by the Infectious Diseases Team. Information regarding HIV testing and the reasons for this audit was given to patients on admission. A standardised proforma documenting data including patients' demographic, reasons for non-consent and its acceptability was completed. A fourth generation blood test (Enzygnost HIV Integral II) was used with the aim of providing results within 36 hours.

## Summary of results

586 patients were considered for testing (16% of total admissions during audit period). 396(67.5%) consented (mean age 59.3 with 42% >age 65). Tests were not performed on 190 (mean age 72.6 with 75% >age 65). 108(57%) of these lacked capacity to consent. 82(43%) refused testing with 59% believing they were not at risk and only 5% believing EAU was an inappropriate place to test. Patients that were approached but not tested were on the average 13.3 years older than those who consented (p<0.001). There were two new HIV diagnoses. Both had PCP (one from Zambia, one MSM). Point prevalence of HIV in EAU was ~5 per 1000. 100% of results were available within 36 hours.

## Conclusions

This pilot study demonstrates that HIV testing in an EAU setting is acceptable to the majority of patients and providing results within 36 hours of admission is feasible. Factors limiting testing include stigma (patients/staff), restrictions on time and misperceptions about what an HIV test entails. It is likely to be more cost effective to offer testing in an EAU setting to those in high risk groups or presenting with indicator diseases. An 'HIV test indicated?' prompt on an admissions proforma may be a useful reminder for staff to consider offering testing and normalise HIV testing in a general medical setting.
